# Inequality in housing transitions during cognitive decline

**DOI:** 10.1371/journal.pone.0282329

**Published:** 2023-04-12

**Authors:** Sarah L. Mawhorter, Rachel Z. Wilkie, Jennifer A. Ailshire

**Affiliations:** 1 Department of Planning and Department of Economic Geography, Faculty of Spatial Sciences, University of Groningen, Groningen, The Netherlands; 2 Leonard Davis School of Gerontology, University of Southern California, Los Angeles, California, United States of America; University of Georgia, UNITED STATES

## Abstract

Independent living can become challenging for people experiencing cognitive decline. With reduced functioning and greater care needs, many people with dementia (PWD) may need to move to another home with better safety features, move to live closer to or with relatives who can provide care, or enter a nursing home. Housing plays a key role in supporting quality of life for both PWD and their caregivers, so the ability to move when needed is crucial for their well-being. Yet the substantial costs of moving, housing, and care mean that PWD with limited financial resources may be unable to afford moving, exacerbating inequalities between more and less advantaged PWD. Emerging qualitative research considers the housing choices of PWD and their caregivers, yet little is known on a broader scale about the housing transitions PWD actually make over the course of cognitive decline. Prior quantitative research focuses specifically on nursing home admissions; questions remain about how often PWD move to another home or move in with relatives. This study investigates socioeconomic and racial/ethnic disparities in the timing and type of housing transitions among PWD in the United States, using Health and Retirement study data from 2002 through 2016. We find that over half of PWD move in the years around dementia onset (28% move once, and 28% move twice or more) while 44% remain in place. Examining various types of moves, 35% move to another home, 32% move into nursing homes, and 11% move in with relatives. We find disparities by educational attainment and race/ethnicity: more advantaged PWD are more likely to move to another home and more likely to enter a nursing home than less advantaged groups. This highlights the importance of providing support for PWD and their families to transition into different living arrangements as their housing needs change.

## Introduction

Living independently can become challenging for people experiencing cognitive decline. Maintenance problems [1, 2] and financial mistakes [3] can make it hard to manage a home even during the early stages of dementia. As dementia progresses, independent living can become unsafe, absent home modifications to prevent accidental self-harm or getting lost [4]. With reduced functioning and greater care needs, many people with dementia (PWD) may need to move to another home with better safety features, move to live closer to or with caregivers, or enter a nursing home [5–7].

At the same time, there can be good reasons for PWD to remain in their longtime homes. Most older adults, including those with dementia, hope to remain in their homes as they age [8, 9]. Adapting a new environment can be especially difficult for those with memory problems [10]. A familiar house and neighborhood can offer continuity, as well as social engagement, support, and a sense of belonging [10–12].

Whether they move or remain in place, housing plays a key role in the quality of life of both PWD and their caregivers [9, 13, 14], making the decision about where to live one of the most consequential choices they must make. Housing decisions can be emotionally fraught and logistically complex for PWD and their families, as desires for comfort in a well-known environment must be weighed alongside the practical demands of dementia care [4, 11, 15]. PWD with a spouse or children living nearby who can provide care may be able to remain in their homes; otherwise they may need to move in with relatives or enter a nursing home [6, 16, 17].

Housing decisions are made all the more difficult because of the substantial costs of moving, housing, care, and nursing homes [18]. PWD with limited financial resources may not be able to afford to move, potentially leaving them in less-than-ideal situations. Conversely, they may not be able to afford to stay in their homes, facing the loss of a familiar place to live and even housing insecurity in some cases. Whether PWD are unable to remain in their homes or unable to move when needed, inadequate housing can lead to daily discomfort and stress, and make it harder for vulnerable PWD to access critical resources and care. In this way, housing disparities may compound disparities in dementia incidence and dementia care [18], exacerbating the inequality between more and less advantaged PWD.

Understanding the patterns of PWDs’ housing choices is necessary to assess their personal and societal impacts, as well as to develop policies that support PWD and their families. Most prior studies in this area focus specifically on nursing home admission, and provide conflicting evidence on whether more socioeconomically advantaged PWD are more likely to be institutionalized [12, 19–21]. An emerging body of research is starting to build knowledge around housing preferences and factors influencing the housing choices of PWD and their caregivers, mainly through in-depth qualitative studies, with some synthesis in review articles [4, 6, 9, 10, 13, 17, 22, 23]. Yet large-scale studies that examine the various settings where PWD live are scarce, and even less is known about PWDs’ actual housing transitions over the course of cognitive decline. Considering the full range of housing transitions, it is still an open question whether more advantaged PWD may remain longer in their homes, or whether they may move earlier during cognitive decline.

This paper begins to fill this gap in knowledge on dementia and housing by investigating socioeconomic and racial/ethnic disparities in the timing and type of housing transitions among PWD in the United States, using Health and Retirement study data from 2002 through 2016. Our analysis addresses two sets of research questions, in order to understand the general patterns and then inequalities in housing transitions among PWD. First, what share of PWD move, what types of moves do they make, and what is the timing of their moves in relation to dementia onset? Second, how do PWDs’ housing transitions vary across sociodemographic groups, focusing on disparities by race/ethnicity and educational attainment?

This research is relevant for understanding the social and economic costs of Alzheimer’s disease and other dementias, currently affecting around seven million older adults in the US [24–26], a population that is expected to more than double by 2050 [26–28]. The findings highlight the importance of providing support for PWD and their families to transition into different living arrangements as their housing needs change.

## Methods

To address our research questions, we use data from the Health and Retirement Study (HRS), a nationally representative, longitudinal study of adults over age 50 in the United States that began in 1992 and has interviewed respondents and their spouses approximately every two years since [29, 30]. The HRS collects detailed information on respondents’ health, physical and cognitive capacity, participation in daily activities, living arrangements, economic status, and well-being. The University of Southern California Institutional Review Board gave ethical approval for this secondary data analysis.

The HRS collects information from respondents, proxies, and interviewers to identify cognitive impairment, which is widely recognized as an acceptable threshold to define dementia cases in the population [24, 31, 32]. Respondents are administered the Telephone Instrument for Cognitive Status (TICS) to evaluate their memory, orientation, and executive function [33, 34]. Based on their responses, as well as assessments from proxies and interviewers, the Langa-Weir Classification of Cognitive Function classifies individuals as having cognitive function consistent with dementia [35]. This classification approach has good predictive ability, as evidenced from comparisons with dementia diagnosis by a consensus panel of experts from neuropsychiatric assessments [36]. We define the onset of dementia as the first survey wave when a respondent is classified as having dementia, as long as their cognitive functioning does not return to normal in subsequent waves.

### Sample

Because we are interested in housing transitions around the time of dementia onset, we limit our sample to PWD who were classified as having dementia onset after they entered the survey (N = 3,896). In order to understand transitions following dementia onset, we restrict the sample to respondents who lived independently beforehand, excluding those who lived in nursing homes (n = 53) or with relatives (n = 78) prior to dementia onset. We likewise exclude those missing information on housing status before dementia onset (n = 222). Our sample does include renters and mobile home residents, vulnerable populations who are often left out of housing-related studies of older adults. We restrict the sample to exclude respondents without information on whether they stayed in place or moved (n = 379), functional limitations (n = 2), or child proximity (n = 34). We further restrict the sample by excluding participants with missing information on race/ethnicity (n = 3); there was no missing information on other characteristics. The final analytic sample consists of N = 3,125 PWD.

We observe whether the PWD in our sample made housing transitions during the two-year periods between HRS survey waves, using data from 2002 through 2016. In order to compare housing transitions over the entire course of cognitive decline (which may occur before the full onset of dementia), we begin observing the PWD respondents in our sample starting up to three survey waves before the recorded onset of dementia, as data is available. This provides a comparison with the housing transitions made before dementia onset. On average, there are four valid between-wave observations (representing eight years) for each of the PWD in the sample, yielding a total of 11,331 observations of potential housing transitions between survey waves. Since this between-wave event analysis involves multiple observations over time for each PWD, we cluster standard errors by respondent.

### Outcome: Housing transitions

Our outcome of interest is whether a respondent (1) remained in the same home, (2) moved to a different home in the community, (3) moved in with relatives, or (4) entered a nursing home during the two-year periods between consecutive survey waves. By examining all between-wave periods starting before dementia onset onward, we can observe multiple housing transitions of various types for the same PWD. For example a PWD may first sell their home to downsize, then later move to a nursing home. Or they may move to another home to be closer to their children, and eventually move in with their children.

### Timing of housing transitions

Understanding the patterns of housing transition over time can yield insights into the adjustments PWD and their families make as dementia progresses. Several studies specifically examine nursing home admission over time following dementia diagnosis (though we were unable to find any prior studies measuring the timing of PWDs’ housing transitions across various types of moves). Both Joling et al and Mjørud et al find relatively steep rates of nursing home admission in the first four years after diagnosis in the Netherlands and in Norway respectively [19, 37]. To measure the timing of housing transitions, we include a continuous specification of years relative to dementia onset in our model.

### Functional limitations

Functional limitations are highly prevalent in dementia illness, with many PWD experiencing difficulty with the instrumental activities of daily living (IADLs) [38]. The inability to perform IADL skills, such as managing money and medications, can affect a PWD’s ability to live independently in their home and contribute to likelihood of transitioning to a nursing home [38–40]. IADL disabilities can also contribute to decisions to downsize, move closer to relatives, or move in with relatives who can provide informal care [41]. Because of evidence that difficulties with IADLs may compromise the ability to maintain an independent household even before more basic ADL disabilities set in [3], we focus on IADL disability in our analysis. We measure the following five IADLs: some difficulty using the telephone, managing money, shopping for groceries, preparing a hot meal, or taking medications. We classify PWD with three or more IADL disabilities as experiencing severe disability in a given wave, and compare their housing transitions with PWD with zero to two IADL disabilities.

### Demographic and family characteristics

Housing transitions often follow life cycle patterns: even absent dementia, older adults may move or downsize in the years after retirement, and then make further adjustments as their needs continue to change [42–45]. We use broad categories of age (51 to 64, 65 to 74, 75 to 84, and 85 years and older) to avoid collinearity with the more fine-grained timing variable around dementia onset. We also include gender as a control variable, as some prior studies find that women are more likely to be admitted to nursing homes than men, possibly due to their later mortality and the likelihood of outliving their spouses who might have provided care [19, 37]; other studies find no statistically significant differences in nursing home admission between women and men when controlling for some of these factors [12, 20].

Family considerations may also play into housing transitions. Marital status changes such as marriage, separation, divorce, or becoming widowed can trigger moves [46]. PWD who receive care from their spouse may be less likely to move [16, 17]. The proximity of adult children is often relevant to the timing of moves among PWD. Social support can impact the likelihood of nursing home admission [12] and having adult children living nearby consistently reduces the likelihood of moving [47, 48]. We include a categorical measure of child proximity, comparing PWD who have one or more children living within 10 miles, PWD whose children live over 10 miles away, and PWD with no living children.

### Housing tenure

Housing tenure has important implications for housing transitions. Homeowners have greater security of tenure than renters, and so are often able to remain longer in their homes [22, 49]. Prior studies have found that renters have an increased likelihood of transitioning to a nursing home or assisted living [50, 51] and people who live in their homes longer are less likely to move in older age [52]. Older homeowners who have paid off their mortgages bear minimal housing costs, and also may have substantial home equity which can be used to finance moves and/or long-term care [53, 54]. We define housing tenure as owning or renting a residence or living in a mobile home three waves (six years) prior to dementia onset, or two waves or one wave prior if the information is missing for three waves earlier. We define mobile home residence as a separate tenure category since the majority of mobile home residents in the HRS have split housing tenure: they own their mobile home but rent the site.

### Socioeconomic status

We examine how PWD with different socioeconomic backgrounds navigate housing transitions over the course of cognitive decline in order to assess how advantage and disadvantage are related to remaining in place or moving. There are marked socioeconomic differences in the living arrangements of older adults in the United States, even beyond the differences captured in the housing tenure variable. Prior studies have found that lower educated older adults are more likely to live with adult children, and higher educated older adults live further from their children [55]. Additionally, studies in Europe have found that in the general population, people of lower socioeconomic status or with lower educational attainment are more likely to be admitted to nursing homes [56–58]. Research in the US context during the early 1980s found a similar association among PWD more specifically [21]. Yet more recent studies in European contexts have found evidence that PWD with higher educational attainment levels are more likely to live in nursing homes [19, 20]. This conflicting evidence may reflect tensions between inequalities in the need for nursing care and the undesirability of institutionalization versus the privilege of accessing nursing home care when needed, and is likely to be highly context-specific based on the availability, costs, and quality of nursing home arrangements in various countries. We use educational attainment, which is closely linked with financial resources such as income and wealth, as a proxy for socioeconomic status. We define three categories of educational attainment, comparing PWD without a high school degree, high school graduates, and college graduates.

### Race/ethnicity

Race and ethnicity are tightly woven into the US housing system through ongoing processes of discrimination in mortgage lending and real estate markets [59], as well as intergenerational wealth transfers [60, 61]. This can contribute to housing insecurity for disadvantaged groups, and at the same time reduce their access to housing opportunities, making it more difficult to move to another home. Racial inequalities in utilization of nursing home and in-home care have been widely documented; Black or Hispanic PWD often have delayed access to care compared with White and other races/ethnicities [40, 62–64]. Black or Hispanic older adults are also more likely to live with adult children and receive informal care compared to White older adults [41, 55, 63]. The racial/ethnic categories in the HRS allow us to identify White, Black, and Hispanic PWD, as well as those with some other race/ethnicity.

### Analytic strategy

We first estimate the prevalence of housing transitions among PWD. We report both the number and type of moves PWD make starting four years before dementia onset and onward for as long as they are observed in the survey. For these estimates we restrict the sample to PWD whose housing transitions are observed across at least three survey waves, or four years (N = 2,823). Including respondents who are observed over only two waves would inflate the number of respondents counted as remaining in place, since that is the most common outcome. For this same reason, our estimates of how many PWD move are likely to be conservative. We use sample weights to correct for differential survey response and to obtain estimates that are representative of the US older adult population. All analyses are conducted using Stata version 17.

We then use multinomial logistic regression to estimate the relative likelihood of PWD making each type of housing transition between two survey waves as compared with remaining in their homes. This study’s longitudinal approach—observing PWDs’ housing transitions rather than their housing status at any one point in time—avoids many of the pitfalls of endogeneity common in cross-sectional housing studies. One drawback of the housing transitions approach is that it does not adjust for differential mortality rates across groups, which can be better accounted for in housing status approaches by including deceased as a status. Still, we chose the housing transitions approach because it allows us to easily observe multiple events of various types over time. Observing the transitions over each two-year period makes it more straightforward to analyze the complex patterns of PWDs’ housing trajectories without imposing assumptions about the order of moving from one type of housing to another. The housing transitions approach also focuses on the choices PWD and their families must make, as practical and policy-relevant outcomes.

## Results

**Fig 1:**
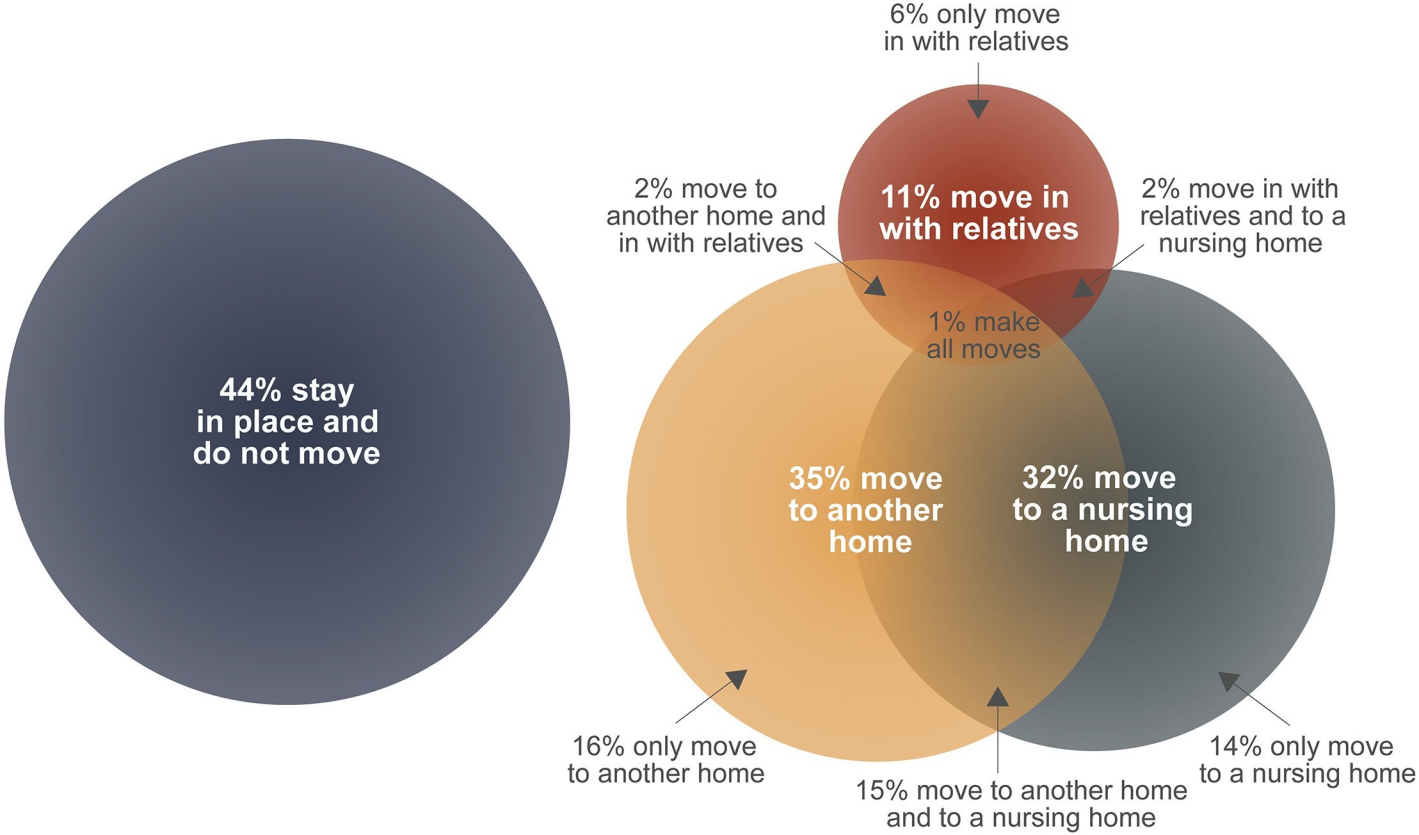
Venn diagram of housing transitions during cognitive decline. Note: Weighted estimates based on PWD in the HRS observed over at least four years between the 2002–2016 survey waves (N = 2,823).

Our descriptive estimates show that over half (56%) of PWD move during the course of cognitive decline. Fig 1 illustrates the types of moves that PWD make: 35% of PWD move to another home that they own or rent, 11% move in with relatives, and 32% move into a nursing home. Since 28% of PWD make multiple moves, there is overlap between these categories. In detail, 16% of PWD move only to another home, and 15% move to another home and into a nursing home. A slightly smaller share (14%) move only into a nursing home. Moving in with relatives is less common: 6% move only in with relatives, 2% move to another home and also in with relatives, and 2% move into a nursing home and also in with relatives. Only 1% make all three types of moves.

### Multivariate analysis

To understand the patterns behind these moves, we turn to multivariate analysis. Table 1 shows the characteristics of the 11,331 between-wave observations in our analytic sample, which includes 3,125 respondents. On average over the two-year periods between survey waves, 75% remained in the same home, 14% moved to another home that they owned or rented, 3% moved in with relatives, and 8% moved into a nursing home. The timing of observations is spread fairly evenly across the survey waves during cognitive decline, peaking slightly in the dementia onset year since all respondents in the sample were observed then. There are slightly fewer observations in earlier and later years due to missing information and because some entered the survey only two years before dementia onset and some left the survey or died in the years following dementia onset.

**Table 1:** Sample characteristics.

Data: Health and Retirement Study 2002–2016			
Respondents: 3,125; Observations: 11,331		Prop	*(SE)*
**Housing transitions**	Stayed	.745	*(.007)*
observed at the end of	Moved to another home	.144	*(.006)*
each two-year period	Moved in with relatives	.034	*(.002)*
between survey waves	Moved into nursing home	.078	*(.003)*
**Years from dementia onset**	4 years before	.135	*(.003)*
at end of two-year period	2 years before	.174	*(.003)*
	Survey year of dementia onset	.224	*(.003)*
	2 years after	.182	*(.002)*
	4 years after	.121	*(.003)*
	6+ years after	.164	*(.006)*
**IADL disability**	Severe IADL disability	.317	*(.007)*
at end of two-year period			
**Age**	51–64	.121	*(.009)*
at end of two-year period	65–74	.211	*(.007)*
	75–84	.360	*(.009)*
	85+	.308	*(.009)*
**Gender**	Female	.618	*(.008)*
**Marital status**	Never married	.039	*(.005)*
at end of two-year period	Married	.415	*(.010)*
	Separated, divorced, or widowed	.546	*(.012)*
**Child proximity**	No living children	.077	*(.007)*
at start of two-year period	All children live >10 miles away	.335	*(.008)*
	A child lives <10 miles away	.588	*(.009)*
**Housing tenure**	Owned home	.685	*(.013)*
before dementia onset	Rented home	.251	*(.012)*
	Mobile home	.065	*(.008)*
**Education**	No high school degree	.468	*(.013)*
	High school graduate	.444	*(.012)*
	College graduate	.087	*(.007)*
**Race/ethnicity**	White	.664	*(.020)*
	Black	.183	*(.013)*
	Hispanic	.125	*(.014)*
	Other	.028	*(.007)*

Notes: HRS survey weights applied. Housing transitions are observed over the two-year periods between survey waves. Time-varying characteristics years from dementia onset, disability, age, and marital status are measured at the end of each two-year period. For example, a respondent married in the 2014 survey and divorced by the 2016 survey is counted as divorced for the moving period between 2014 and 2016. The exception is child proximity, measured at the beginning of each two-year period since it may serve as a motivation for a subsequent move.

In nearly a third of observations (32%), respondents were classified with severe IADL disability. Because our observations are linked to the timing of dementia onset, most (67%) occur when respondents are aged 75 years or older. Women represent the majority of observations (62%) due to longer life spans among women and their higher prevalence of dementia. The timing of dementia onset also accounts for the relatively large proportion of observations (55%) when respondents are separated, divorced, or widowed. Most of the remaining observations (42%) represent married respondents, and a small share (4%) never married. In the majority of observations (59%) respondents have at least one child living within ten miles, in a third (34%) respondents’ children live more than ten miles away, and in a small minority (8%) respondents have no living children.

The large majority of observations (69%) represent PWD who owned their homes before dementia onset, another quarter (25%) represent PWD who rented their homes, and the remaining 7% represent PWD who lived in mobile homes. Nearly half (47%) of observations are of PWD without a high school degree, another 44% are of high school graduates, and only 9% are of college graduates. In terms of race/ethnicity, 66% of observations represent White respondents, 18% represent Black respondents, 13% represent Hispanic respondents, and 3% represent those with another race/ethnicity.

Table 2 shows the relative risk ratios from a multinomial logistic regression estimating PWDs’ likelihood of moving to another home, moving in with relatives, or moving to a nursing home as opposed to staying in place. Relative risk ratios below one indicate a lower likelihood of making a housing transition compared with the reference group, and relative risk ratios above one indicate a higher likelihood of making a housing transition.

**Table 2:** Relative risk ratios from multinomial logistic regression estimating PWDs’ likelihood of making housing transitions.

Health and Retirement Study 2002–2016	Moved to another home				Moved in with relatives				Moved to a nursing home			
Respondents: 3,125; Observations: 11,331												
	RRR		*(SE)*	RRR			*(SE)*	RRR			*(SE)*
**Years from dementia onset**	.979		*(.081)*	1.621		**	*(.262)*	2.639		***	*(.488)*
Quadratic term	1.021		*(.020)*	.912		*	*(.034)*	.862		***	*(.032)*
Cubic term	.999		*(.001)*	1.005		*	*(.002)*	1.006		**	*(.002)*
**Severe IADL disability**	2.746	***	*(.231)*	2.319		***	*(.306)*	11.145		***	*(1*.*267)*
**Age**, ref. 51–64											
65–74	.810		*(.124)*	.912			*(.264)*	1.290			*(.391)*
75–84	.729	*	*(.111)*	1.954		*	*(.522)*	1.915		*	*(.543)*
85+	.813		*(.131)*	1.935		*	*(.530)*	1.968		*	*(.563)*
**Female**, ref. male	.990		*(.090)*	1.028			*(.162)*	.947			*(.093)*
**Marital status**, ref. married											
Never married	1.279		*(.284)*	3.655		***	*(1*.*269)*	1.370			*(.425)*
Separated, divorced, or widowed	2.328	***	*(.220)*	3.556		***	*(.598)*	2.169		***	*(.232)*
**Child proximity**, ref. children live >10 miles											
A child lives <10 miles away	.796	**	*(.064)*	.711		**	*(.091)*	.682		***	*(.062)*
No living children	1.028		*(.182)*	.660			*(.179)*	1.058			*(.192)*
**Housing tenure pre-onset**, ref. owner											
Rented home before dementia onset	2.696	***	*(.256)*	1.348			*(.210)*	1.814		***	*(.177)*
Mobile home before dementia onset	1.465	*	*(.249)*	1.005			*(.262)*	1.225			*(.208)*
**Education**, ref. no high school degree											
High school graduate	1.495	***	*(.141)*	.810			*(.116)*	1.428		***	*(.130)*
College graduate	1.671	***	*(.258)*	.647			*(.176)*	1.307			*(.192)*
**Race/ethnicity**, ref. White											
Black	.528	***	*(.061)*	1.100			*(.181)*	.447		***	*(.059)*
Hispanic	.519	***	*(.082)*	1.500		*	*(.279)*	.269		***	*(.053)*
Other	.664		*(.163)*	.997			*(.399)*	.672			*(.240)*

Note: HRS survey weights applied; standard errors clustered by respondent;

* p < .05,

** p < .01,

*** p < .001

**Fig 2:**
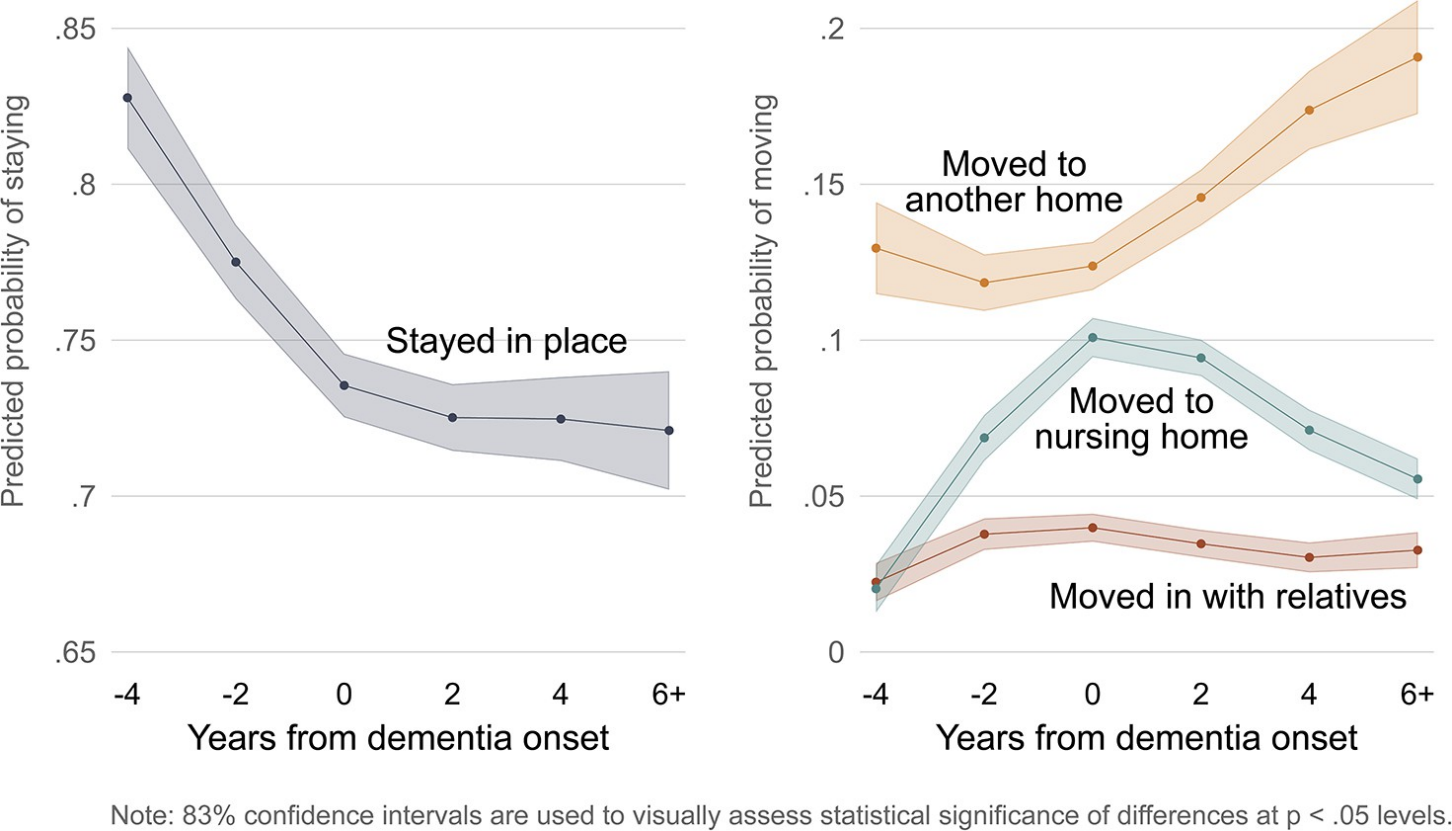
Predicted probabilities of PWDs’ housing transitions by years from dementia onset. Note: Predicted probabilities of housing transitions from multinomial logistic regression model shown in Table 2. 83% confidence intervals are used to visually assess statistical significance of differences at the p < .05 level.

This model helps us understand the timing of housing transitions among PWD, controlling for other factors. In order to facilitate interpretation of the model estimates, we plot the predicted probabilities of PWD remaining in place or making each of the three types of housing transitions starting four years before dementia onset through six years or more after dementia onset, as shown in Fig 2. At four years before dementia onset, 83% of those who will go on to develop dementia remain in their homes. As time goes on, fewer PWD stay in place, until at two years after dementia onset slightly over 72% of PWD stay in place, and this proportion levels off going forward.

This also means that by two years after dementia onset and onward, over a quarter (26% to 28%) of PWD make some type of housing transition during any given two-year period. Compare this with four years before dementia onset, when an estimated 17% move, and most moves are to another home that they own or rent. Moves to another home increase as the years progress, hovering around 12% or 13% in the years leading up to dementia onset and then rising to 19% by six years after dementia onset. (While the relative risk ratios for years since dementia onset for the outcome of moving to another home do not appear as statistically significant, in a separate model with only a linear term, years since dementia onset has a relative risk ratio of 1.076, statistically significant at the *p* < .001 level. We show the model with the quadratic and cubic terms since they better fit the other two outcomes.)

At four years before dementia onset, only 2% of PWD in our sample move into a nursing home on average. By the time of dementia onset, the average share moving to nursing homes peaks at 10%. The share of PWD moving to nursing homes declines after that point, down to 6% by six years after dementia onset. The proportion who move in with relatives rises from 2% four years before dementia onset to 4% two years before and in the year of dementia onset, then declines slightly to 3% in later periods.

**Fig 3:**
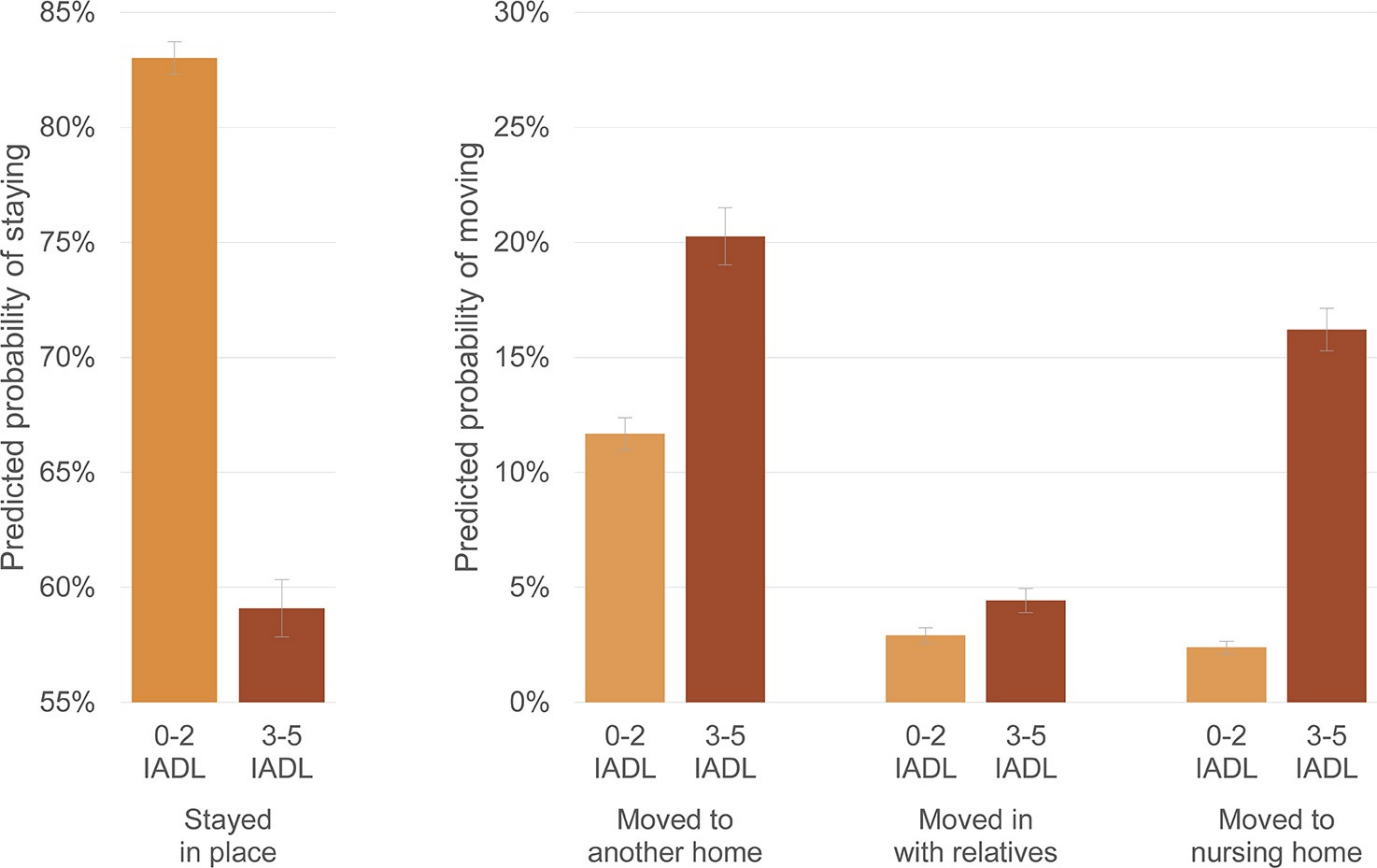
Predicted probabilities of PWDs’ housing transitions by IADL disability. Note: Predicted probabilities of housing transitions from multinomial logistic regression model shown in Table 2. 83% confidence intervals are used to visually assess statistical significance of differences at the p < .05 level.

As expected, IADL disability is strongly related to the likelihood of housing transitions among PWDs. Those with severe IADL disability are 2.746 times as likely to move to another home over a two-year period (*p* < .001), 2.319 times as likely to move in with relatives (*p* < .001), and fully 11.145 times as likely to move to a nursing home (*p* < .001). To understand the magnitude of these differences, Fig 3 graphs the predicted probability of PWD remaining in place or moving by whether they are classified as having severe IADL disability (on 3 to 5 activities) in each wave. Under 60% of PWDs with severe IADL disability remain in the same home over each two-year period, as compared with 83% of those without severe IADL disability. In terms of moves, 20% of PWDs with severe IADL disability move to another home, as compared with around 12% of those without. Between 4% and 5% of PWD with severe IADL disability move in with relatives, as compared with just under 3% of those without. Nursing home moves are even more strongly related to IADL disability: 16% of PWD with severe IADL disability move into nursing homes, while around 2% of those without move into nursing homes.

Taking IADL disability and the years from dementia onset as well as the other factors in the model into account, moves to another home are less strongly related to age among PWD than might be expected. PWD aged 75 to 84 are less likely to move to another home than those aged 51 to 64 (RRR = .729, *p* < .05), yet the other age groups all move at similar rates on average. As expected, moving in with relatives and into nursing homes increase with age. PWD aged 75 to 84 and PWD aged 85 and over are more likely to move in with relatives than PWD in their fifties and early sixties (RRR for ages 75 to 84 = 1.954, *p* < .05; RRR for ages 85 and over = 1.935, *p* < .05). Similarly, older PWD are more likely to move into nursing homes (RRR for ages 75 to 84 = 1.915, *p* < .05; RRR for ages 85 and over = 1.968, *p* < .05). PWDs’ housing transitions do not appear to vary by gender when accounting for these other factors.

Family circumstances also play a role in PWDs’ housing transitions. Compared with married PWD, single PWD who never married are far more likely to move in with relatives (RRR = 3.655, *p* < .001). PWD who are separated, divorced, or widowed are more likely to make all three types of housing transitions as compared with married PWD. Separated, divorced, or widowed PWD are 2.328 times as likely than married PWD to move to another home (*p* < .001), 3.556 times as likely to move in with relatives (*p* < .001), and 2.169 times as likely to move to a nursing home (*p* < .001). Child proximity appears to matter as well. Compared with PWD whose children all live more than ten miles away, those who have a child living within ten miles of their home are less likely to move to another home (RRR = .796, *p* < .01), less likely to move in with relatives (RRR = .711, *p* < .01), and less likely to move into a nursing home (RRR = .682, *p* < .001). PWD without living children do not make housing transitions at statistically different rates than PWD whose children all live over ten miles away.

**Fig 4:**
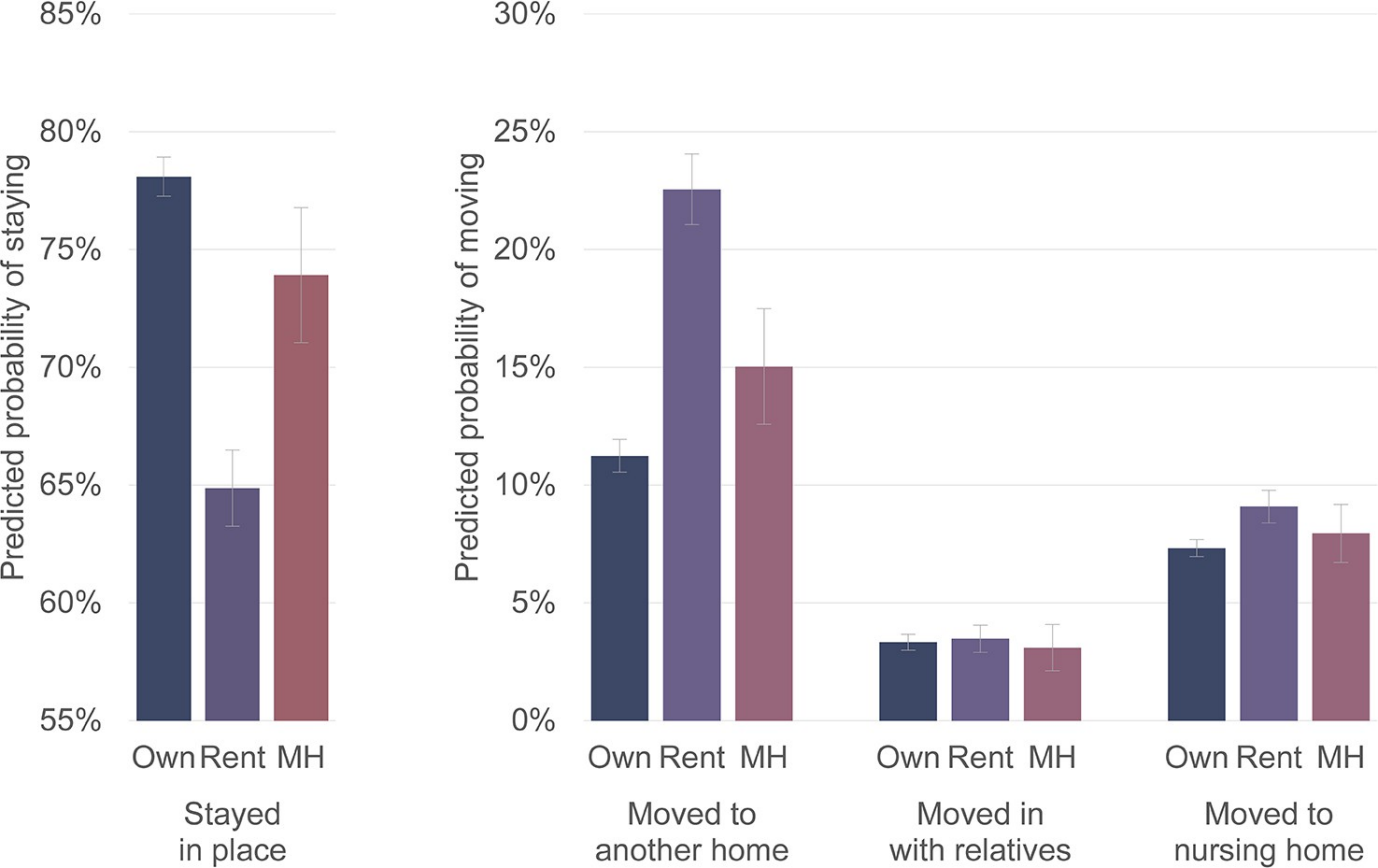
Predicted probabilities of PWDs’ housing transitions by housing tenure before dementia onset. Note: Predicted probabilities of housing transitions from multinomial logistic regression model shown in Table 2. 83% confidence intervals are used to visually assess statistical significance of differences at the p < .05 level.

Housing tenure before dementia onset is also related to later housing transitions, as shown in Fig 4. Compared with PWD who owned their homes, PWD who rented their homes are 2.696 times as likely to move to another home (*p* < .001) and 1.814 times as likely to move to a nursing home (*p* < .001). PWD who lived in mobile homes before dementia onset are 1.465 times as likely to move to another home (*p* < .05). These results are consistent with overall higher residential mobility levels for renters and mobile home residents, who have less security of tenure compared with homeowners.

**Fig 5:**
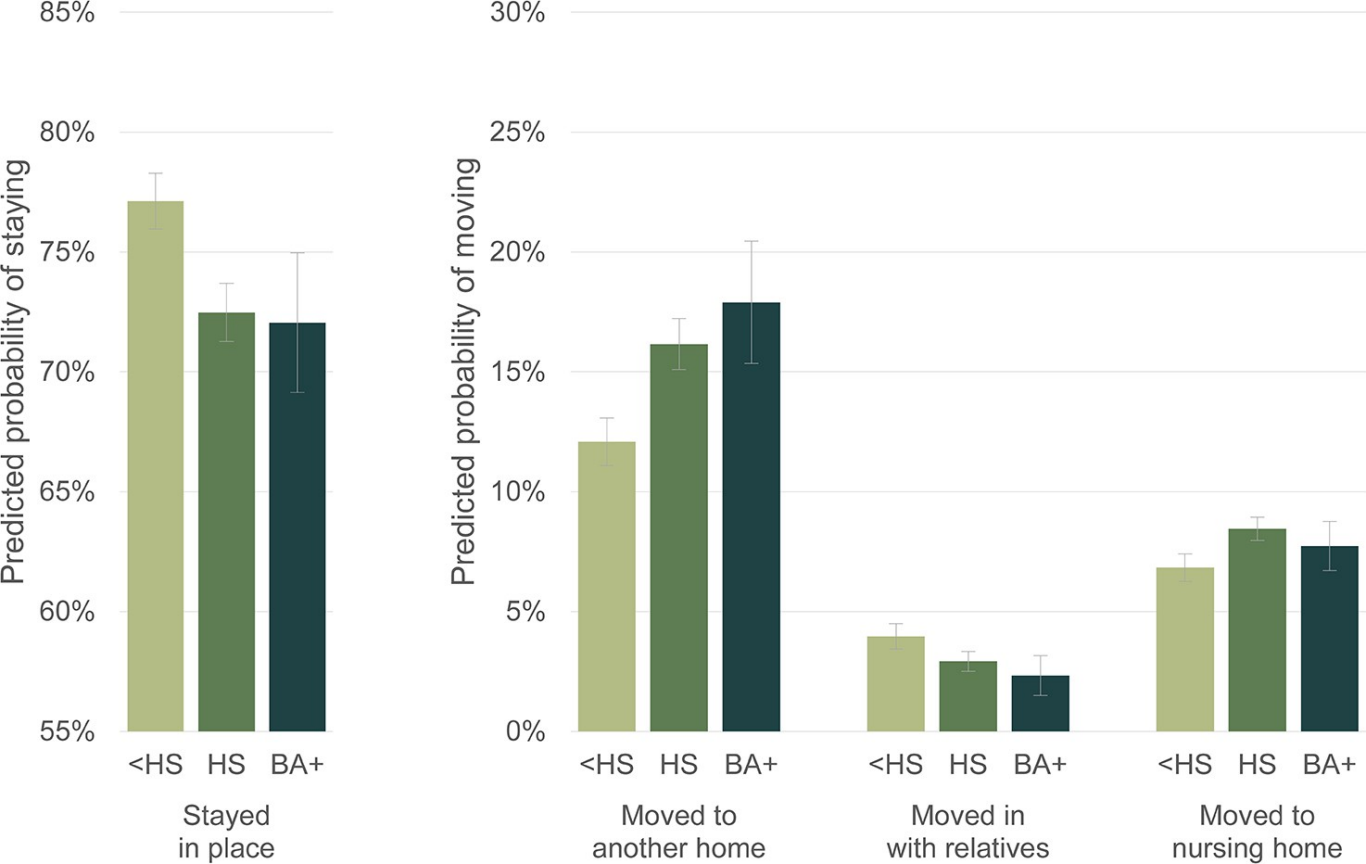
Predicted probabilities of PWDs making housing transitions by education. Note: Predicted probabilities of housing transitions from multinomial logistic regression model shown in Table 2. 83% confidence intervals are used to visually assess statistical significance of differences at the p < .05 level.

Even with these personal, familial, and housing characteristics accounted for, we see distinct patterns of housing transitions across sociodemographic groups. As compared with PWD without a high school degree, PWD who are high school graduates are more likely to move into another home (RRR = 1.495, *p* < .001), and more likely to move into a nursing home (RRR = 1.428, *p* < .001). PWD with college degrees are also more likely to move to another home (RRR = 1.671, *p* < .001). Fig 5 shows PWDs’ predicted probabilities of making housing transitions by educational attainment. This makes it clear that overall, PWD without a high school degree tend to stay in their homes more than PWD who are high school or college graduates.

**Fig 6:**
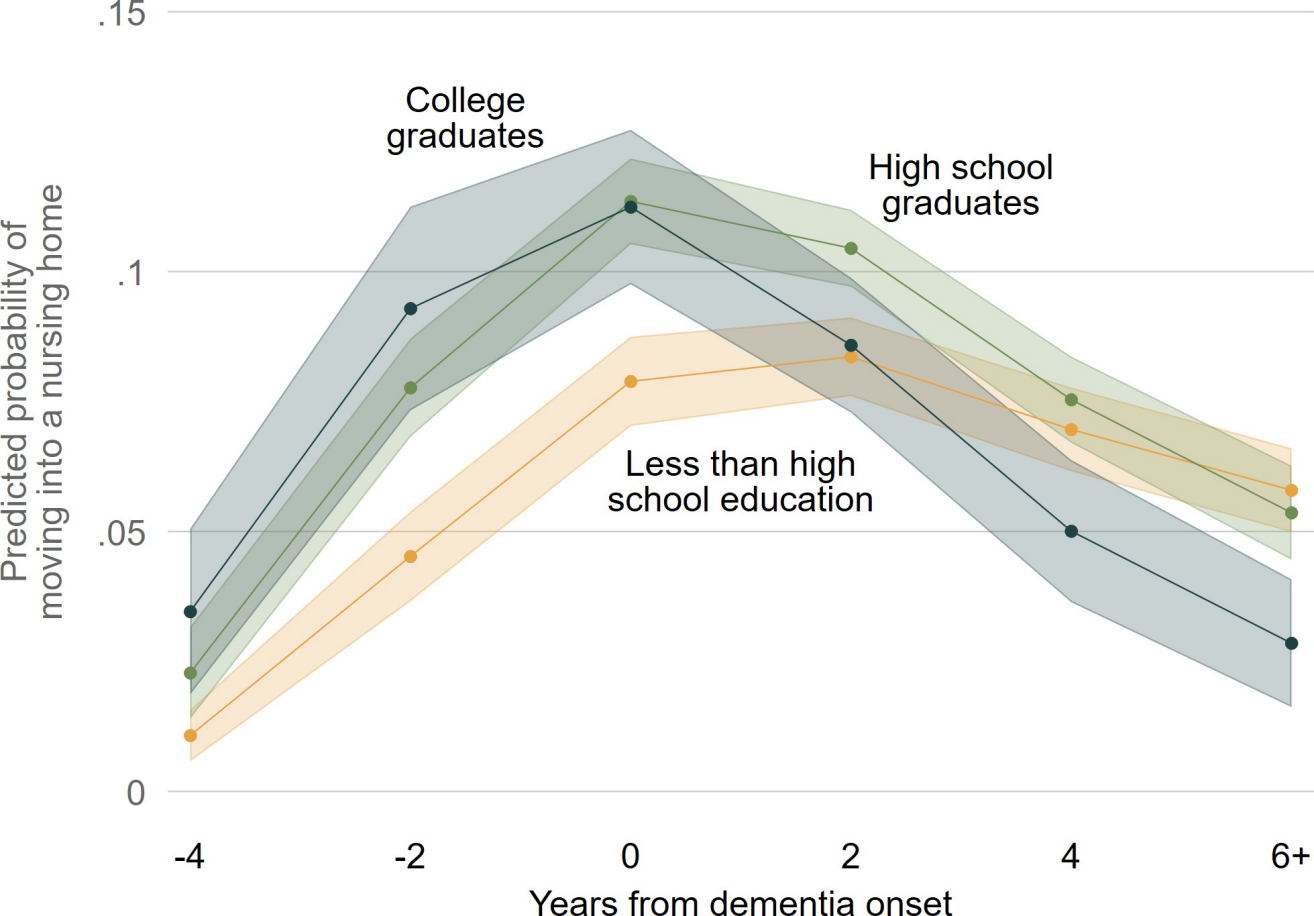
Predicted probabilities of PWDs moving into a nursing home over years from dementia onset interacted with education. Note: Predicted probabilities of moving into a nursing home from multinomial logistic regression model shown in S1 Table. 83% confidence intervals are used to visually assess statistical significance of differences at the p < .05 level.

We then interact education with the years from dementia onset variable, and see that the timing of moves into nursing homes (but not the other types of moves) varies by education level. Fig 6 shows the predicted probabilities of moving to a nursing home by education over time. (The full table of relative risk ratios for this model specification with the added interaction is available in S1 Table.) College and high school graduates more often move into nursing homes early in cognitive decline, up to and including the year of dementia onset. After dementia onset their rate of moving into nursing homes declines, and by four years after onset high school graduates move into nursing homes at similar rates as those without a high school degree, and college graduates move into nursing homes at lower rates.

**Fig 7:**
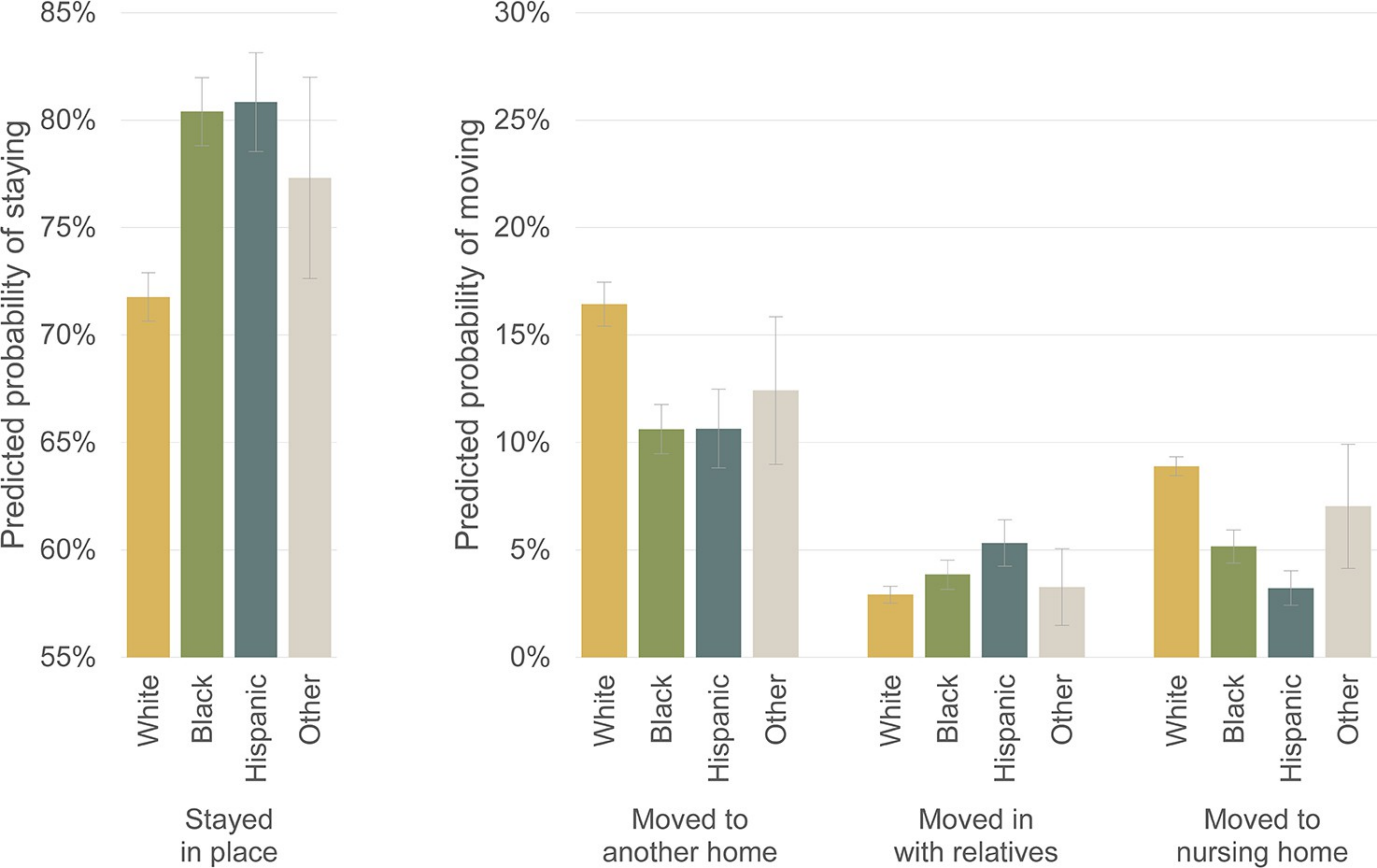
Predicted probabilities of PWDs making housing transitions by race/ethnicity. Note: Predicted probabilities of housing transitions from multinomial logistic regression model shown in Table 2. 83% confidence intervals are used to visually assess statistical significance of differences at the p < .05 level.

The patterns of housing transitions by race/ethnicity, shown in Fig 7, are even more striking. White PWD tend to move more often than Black and Hispanic PWD, usually to other homes and nursing homes. Black PWD are less likely to move to another home than White PWD (RRR = .528, *p* < .001), and less likely to move into a nursing home (RRR = .447, *p* < .001). Similarly, Hispanic PWD are less likely to move into another home than White PWD (RRR = .519, *p* < .001), and far less likely to move into a nursing home (RRR = .269, *p* < .001). On the other hand, Hispanic PWD are more likely to move in with relatives (RRR = 1.500, *p* < .05).

**Fig 8:**
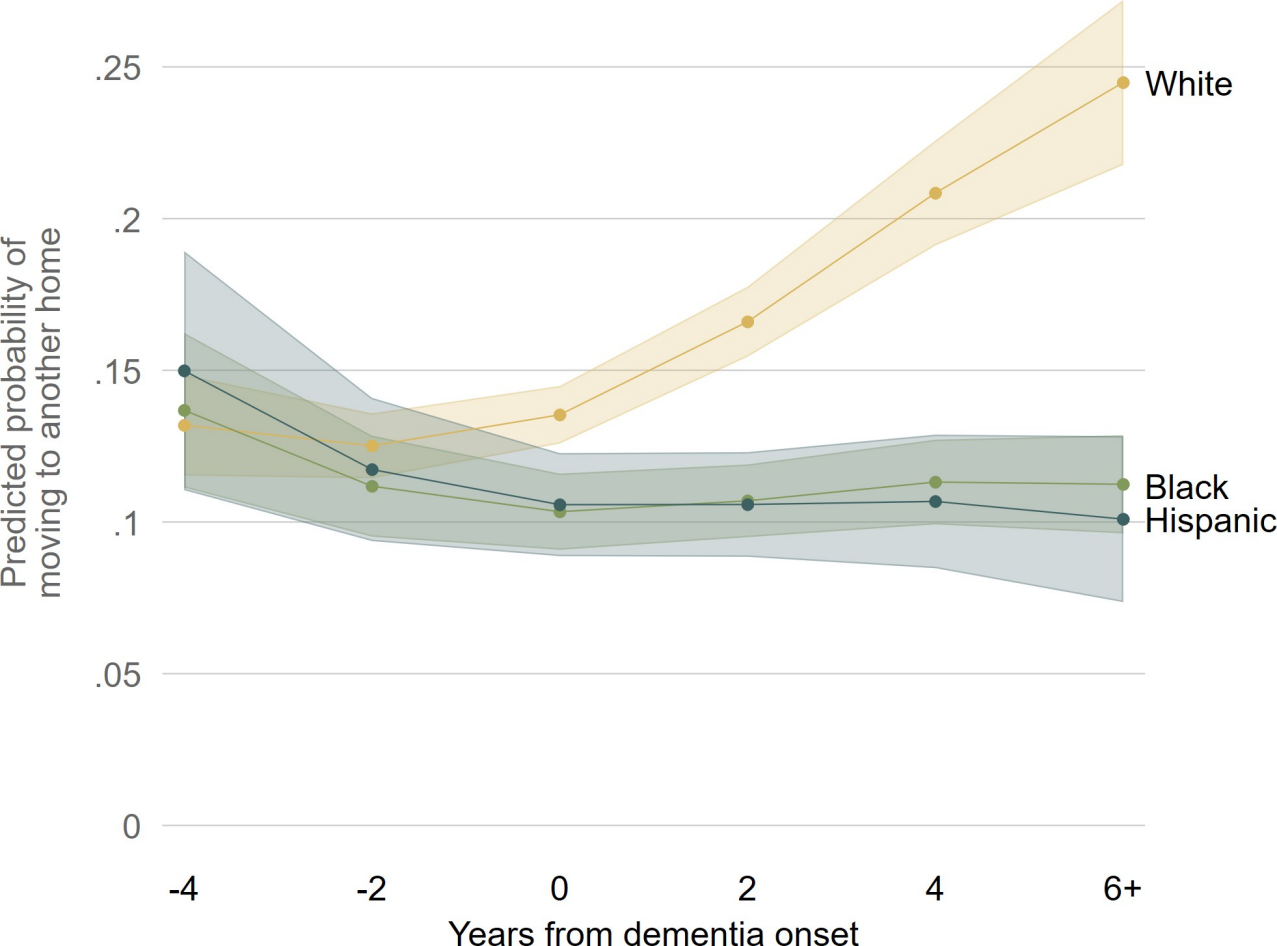
Predicted probabilities of PWDs moving to another home over years from dementia onset interacted with race/ethnicity. Note: Predicted probabilities of moving into another home from multinomial logistic regression model shown in S2 Table. 83% confidence intervals are used to visually assess statistical significance of differences at the p < .05 level.

When race/ethnicity is interacted with timing, a clear pattern emerges for the outcome of moving to another home. As shown in Fig 8, White, Black and Hispanic PWD move to another home at similar rates before dementia onset. (Once again, the full table of relative risk ratios for this interacted model is available in S2 Table.) Starting around the time of dementia onset, White PWD move to another home more frequently, while Black and Hispanic PWD move at level rates throughout the whole course of cognitive decline. By six years after dementia onset, around a quarter of White PWD move to another home, while only around a tenth of Black and Hispanic PWD move to another home.

## Discussion

One clear finding from this research is just how common it is for PWD to move: well over half of PWD make a housing transition at some point during cognitive decline. Over a third of PWDs move to another home, slightly fewer move to nursing homes, and around ten percent move in with relatives. This supports the idea that however strong the desire to remain in a longtime home, the practical demands of living with dementia can make that untenable. The prevalence of multiple moves suggests that as dementia progresses, PWD may need to further adjust their home environment to meet changing needs. With this in mind, housing decisions should be a key consideration discussed as part of treatment plans with the collaboration of caregivers, as discussed by Garvelink et al [6, 65], Adekpedjou et al [11], Campbell et al [23], and Sturge et al [66].

The basic patterns of PWDs’ housing transitions are consistent with expectations. Older PWD move into nursing homes or in with relatives far more often than younger PWD, while moves to another home are slightly less prevalent among older PWD. Married PWD and those with children living nearby tend to remain in their homes rather than moving, which likely reflects the availability of family caregivers for older adults.

Moves in with relatives and into nursing homes peak around the time of dementia onset, which may indicate a sudden greater need for intensive care. Moves to another home in the community increase after dementia onset. Moving to another home may be motivated by a desire to live closer to caregivers, health care services, or other amenities that can support PWD [5, 6, 67, 68]. Another home may have advantages in terms of home modifications or other features such as single floor living which might improve the living environment for PWD and their partner [4, 5, 7]. Some PWD may downsize to a smaller home or rental apartment, whether to avoid home maintenance burdens [1, 2] or for financial reasons such as extracting home equity to cover caregiving costs [53, 69]. Still, we find that PWD who owned their homes before dementia onset are more likely to remain in their homes than PWD who rented their homes or lived in mobile homes. This finding is consistent with research showing the relative residential instability of renting and living in a mobile home as compared with conventional homeownership [70–72].

We see clear disparities in PWDs’ housing transitions across education levels and race/ethnicity. More highly educated PWD have a greater tendency to move either to another home or to a nursing home than those without high school degrees. More educated PWD move into nursing homes earlier during cognitive decline. Compared with White PWD, Black and Hispanic PWD are more likely to remain in place, especially as cognitive decline proceeds. These findings are consistent with other studies finding that low-income and Black older adults are more likely to delay entry into both home and residential care [63, 73]. Delays in moves by Black and Hispanic PWD could be attributed to cultural differences in caregiving practices between White, Black, and Hispanic older adults; Black and Hispanic PWD may delay moves to a nursing home due to placing greater value on family-based home care [63, 74]. Furthermore, less advantaged PWD and their families may not have the resources to move [18], raising concerns that they may be stuck in inadequate housing situations.

A key limitation of this study (and avenue for further research) is that it does not directly examine the financial context and implications of PWDs’ housing transitions. Our findings lead to questions about whether PWD who owned their homes before dementia onset sell them to finance dementia care. Future research in this vein should consider housing affordability, housing wealth, and other factors known to play into moving decisions among older adults more generally.

While there is ample room to build on this research, this paper contributes descriptive findings that lay a groundwork for understanding PWDs’ housing transitions. To our knowledge, this is the first large-scale quantitative study to consider the full range of housing alternatives for PWD together, from various living situations in the community through nursing homes. Understanding these housing decisions matters because they are critical for the well-being of PWD and their caregivers. In aggregate, PWDs’ housing and care arrangements contribute to the overall economic costs of dementia, which fall to Medicare and Medicaid as well as PWD and their families [25, 75]. The expense of both housing and care are especially onerous for those with limited financial resources, exacerbating existing socioeconomic inequalities [76]. The results of this initial analysis suggest the importance of providing support for PWD and their families to help them transition into different living arrangements as their housing needs change.

## Supporting information

S1 Table Relative risk ratios from multinomial logistic regression estimating PWDs’ likelihood of making housing transitions, with education by timing interaction. (PDF)

S2 Table Relative risk ratios from multinomial logistic regression estimating PWDs’ likelihood of making housing transitions, with race/ethnicity by timing interaction. (PDF)
