# Supplementary material for: Inequality in housing transitions during cognitive decline
Source: PLoS One. 2023 Apr 12;18(4):e0282329. doi: 10.1371/journal.pone.0282329 (PMC10096249; doi:10.1371/journal.pone.0282329)
Supplement: S1 Table — (PDF) [file pone.0282329.s001.pdf]

S1 Table. Relative risk ratios from multinomial logistic regression estimating PWDs' likelihood of making housing transitions, with education by timing interaction

Health and Retirement Study 2002-2016<br>Respondents: 3,125; Observations: 11,331	Moved to another home		Moved in with relatives		Moved to a nursing home	
	RRR	*(SE)*	RRR	*(SE)*	RRR	*(SE)*
**Years from dementia onset**	.963	*(.081)*	1.571**	*(.256)*	2.880***	*(.545)*
Quadratic term	1.020	*(.020)*	.911*	*(.034)*	.862***	*(.032)*
Cubic term	.999	*(.001)*	1.005*	*(.002)*	1.006**	*(.002)*
**Severe IADL disability**	2.747***	*(.232)*	2.288***	*(.302)*	11.207***	*(1.270)*
**Age**, ref. 51-64						
65-74	.810	*(.124)*	.916	*(.265)*	1.286	*(.391)*
75-84	.733*	*(.111)*	1.978*	*(.525)*	1.905*	*(.542)*
85+	.816	*(.132)*	1.963*	*(.536)*	1.953*	*(.560)*
**Female**, ref. male	.991	*(.090)*	1.027	*(.162)*	.955	*(.094)*
**Marital status**, ref. married						
Never married	1.280	*(.286)*	3.685***	*(1.270)*	1.375	*(.432)*
Separated, divorced, or widowed	2.330***	*(.221)*	3.546***	*(.597)*	2.185***	*(.234)*
**Child proximity**, ref. children live >10 miles						
A child lives <10 miles away	.792**	*(.064)*	.709**	*(.091)*	.683***	*(.062)*
No living children	1.026	*(.182)*	.657	*(.177)*	1.055	*(.192)*
**Housing tenure pre-onset**, ref. owner						
Rented home before dementia onset	2.700***	*(.257)*	1.349	*(.210)*	1.819***	*(.178)*
Mobile home before dementia onset	1.471*	*(.253)*	1.009	*(.263)*	1.220	*(.210)*
**Education**, ref. no high school degree						
High school graduate	1.183	*(.183)*	.564*	*(.141)*	2.230***	*(.513)*
College graduate	1.759*	*(.440)*	.521	*(.275)*	3.889***	*(1.430)*
High school grad × years from onset	1.043	*(.026)*	1.071	*(.043)*	.932*	*(.033)*
College graduate × years from onset	.978	*(.044)*	1.038	*(.098)*	.814**	*(.054)*
**Race/ethnicity**, ref. White						
Black	.528***	*(.061)*	1.103	*(.181)*	.438***	*(.059)*
Hispanic	.521***	*(.082)*	1.508*	*(.280)*	.265***	*(.053)*
Other	.661	*(.163)*	1.002	*(.401)*	.660	*(.233)*

*Note: HRS survey weights applied; standard errors clustered by respondent; * $p < .05$, ** $p < .01$, *** $p < .001$*
