# Supplementary material for: Inequality in housing transitions during cognitive decline
Source: PLoS One. 2023 Apr 12;18(4):e0282329. doi: 10.1371/journal.pone.0282329 (PMC10096249; doi:10.1371/journal.pone.0282329)
Supplement: S2 Table — (PDF) [file pone.0282329.s002.pdf]

S2 Table. Relative risk ratios from multinomial logistic regression estimating PWDs' likelihood of making housing transitions, with race/ethnicity by timing interaction

Health and Retirement Study 2002-2016<br>Respondents: 3,125; Observations: 11,331	Moved to another home		Moved in with relatives		Moved to a nursing home	
	RRR	*(SE)*	RRR	*(SE)*	RRR	*(SE)*
**Years from dementia onset**	1.006	*(.084)*	1.636**	*(.266)*	2.674***	*(.494)*
Quadratic term	1.020	*(.020)*	.911*	*(.034)*	.861***	*(.032)*
Cubic term	.999	*(.001)*	1.005*	*(.002)*	1.007**	*(.002)*
**Severe IADL disability**	2.697***	*(.229)*	2.302***	*(.302)*	11.066***	*(1.257)*
**Age**, ref. 51-64						
65-74	.843	*(.127)*	.908	*(.263)*	1.296	*(.393)*
75-84	.770	*(.116)*	1.952*	*(.519)*	1.932*	*(.550)*
85+	.863	*(.138)*	1.944*	*(.531)*	2.000*	*(.573)*
**Female**, ref. male	1.009	*(.092)*	1.035	*(.164)*	.956	*(.094)*
**Marital status**, ref. married						
Never married	1.310	*(.291)*	3.660***	*(1.277)*	1.376	*(.425)*
Separated, divorced, or widowed	2.292***	*(.216)*	3.561***	*(.597)*	2.154***	*(.230)*
**Child proximity**, ref. children live >10 miles						
A child lives <10 miles away	.795**	*(.065)*	.712**	*(.091)*	.682***	*(.062)*
No living children	1.031	*(.182)*	.664	*(.180)*	1.065	*(.194)*
**Housing tenure pre-onset**, ref. owner						
Rented home before dementia onset	2.716***	*(.260)*	1.348	*(.211)*	1.818***	*(.178)*
Mobile home before dementia onset	1.453*	*(.249)*	1.001	*(.261)*	1.220	*(.208)*
**Education**, ref. no high school degree						
High school graduate	1.497***	*(.141)*	.814	*(.116)*	1.429***	*(.130)*
College graduate	1.709***	*(.263)*	.658	*(.179)*	1.325	*(.195)*
**Race/ethnicity**, ref. White						
Black	1.029	*(.190)*	.985	*(.301)*	.644	*(.197)*
Hispanic	1.199	*(.303)*	2.249*	*(.766)*	.609	*(.299)*
Other	.848	*(.378)*	.776	*(.593)*	.261	*(.286)*
Black × years from onset	.893***	*(.024)*	1.013	*(.049)*	.940	*(.043)*
Hispanic × years from onset	.865***	*(.036)*	.932	*(.047)*	.871	*(.069)*
Other × years from onset	.958	*(.057)*	1.037	*(.110)*	1.142	*(.155)*

*Note: HRS survey weights applied; standard errors clustered by respondent; * p < .05, ** p < .01, *** p < .001*
